# Upregulation of PSMD4 gene by hypoxia in prostate cancer cells

**DOI:** 10.3906/biy-2002-71

**Published:** 2020-10-13

**Authors:** Sümeyye AYDOĞAN TÜRKOĞLU, Gizem DAYI, Feray KÖÇKAR

**Affiliations:** 1 Department of Molecular Biology and Genetics, Faculty of Science and Literature, University of Balıkesir, Balıkesir Turkey; 2 Department of Biology, Faculty of Science and Literature, University of Balıkesir, Balıkesir Turkey

**Keywords:** PSMD4, hypoxia, transcriptional regulation, prostate cancer, endothelial cells

## Abstract

Ubiquitin-proteasome pathways have a crucial role in tumor progression. PSMD4 (Rpn10, 26S proteasome non-ATPase subunit 4), which is a subunit of the regulatory particle, is a major ubiquitin (Ub) receptor of 26S proteasome. PSMD4 overexpression has been observed in colon carcinoma, hepatocellular carcinoma, and breast cancer. In this work, we elucidated the effect of hypoxia on PSMD4 gene expression in prostate cancer cells (PC3). Chemically mimicked hypoxia drastically upregulated PSMD4 gene expression at both mRNA and protein levels. Transient transfection experiments indicated that all promoter fragments were active in PC3 cells. Hypoxia increased transcriptional activity of all PSMD4 promoter constructs. EMSA analysis shows that HIF-1a transcription factor binds to the hypoxia response element (HRE) present within the –98/+52 region of PSMD4 promoter. We also used human umbilical vein endothelial cell (HUVEC) as a different cell model, in which increased PSMD4 expression was seen only at 24 h. The increased expression of the PSMD4 level in the PC3 cell line was not parallel to the expression in hypoxic HUVEC.

## 1. Introduction

In cancer progression, microcommunication between cancer cells and their microenvironment is important. When the tumor cells do not get enough oxygen, they become adaptive to hypoxic conditions by causing various genetic changes to occur without causing cell death. Hypoxia has been shown to play a role in the development of the resistance to radiation and chemotherapeutic treatment in various tumor types (Zhong et al., 1999; Harris 2002; Koyasu et al., 2018).

Ubiquitin-proteasome pathways play a crucial role in the cellular process by regulating the expression of key proteins involved in oncogenesis and cell cycle progression (Shen et al., 2013; Chen et al., 2016). 26S proteasomes catalyze most of the protein degradation in growing mammalian cells. PSMD4 (Rpn10, 26S proteasome non-ATPase subunit 4) and Rpn13, which are 2 subunits of regulatory particles, are major ubiquitin (Ub) receptors of 26S proteasome (Collins and Goldberg, 2017). The PSMD4 gene encodes a ubiquitin-binding protein, S5a, which serves the 19S regulatory complex and exists in soluble form in the cytosol. The PSMD4 gene also encodes ASF (antisecretory factor) and angiocidin. The ASF protein is similar in sequence to S5a but is quite different in function. It inhibits intestinal fluid induced by cholera toxin. Angiocidin is a protein that is overexpressed in various solid tumors and tumor-capillary endothelial cells. Angiocidin is antiangiogenic and inhibits tumor growth (Benaroudj et al., 2003). The carboxy-terminal region of angiocidin carries 3 additional amino acids, unlike the other 2 proteins, S5a and ASF (Nelson et al., 2000).

Numerous studies have shown that PSMD4 plays a significant part in tumor progression. PSMD4 has been found to be overexpressed in specimens of human colon cancer (Cheng et al., 2018). PSMD4 is overexpressed and amplified in breast cancer, correlating with poor survival rates (Fejzo et al., 2017). However, previous studies have also shown that nuclear factor kappa B (NF-κB) and PSMD4 inhibited breast cancer proliferation via epidermal activation growth factor receptors (Godek et al., 2011). In colon cancer cells, increased cNrf2 (nuclear factor-like 2) expression promotes colorectal cancer with more aggressive tumors via upregulating PSMD4 (Lin et al., 2016). PSMD4 increases cell proliferation via regulation of the PTEN/Akt pathways in hepatocellular carcinoma (HCC) cells; in addition, a significant correlation has been found between HIF1a expression and PSMD4 expression in HCC patients. These two parameters are a strong prognosis indicator in HCC (Jiang et al., 2019). In addition, the silencing of PSMD4 decreased cell proliferation in HCC (Chai et al., 2019). Therefore, depending on the type of tumor, PSMD4 could have different roles.

The purpose of the present study is to identify the hypoxic regulation of the PSMD4 gene in a prostate cancer model. Hypoxic conditions were chemically mimicked in the prostate cell line models (PC3) and normal cells (HUVEC cell line) and were verified with the HIF1α mRNA expression level. The cytotoxic effect of hypoxia was analyzed by MTT assay in both cell lines. The levels of mRNA and protein of PSMD4 were compared in normoxic and hypoxic conditions; increased mRNA and protein expression were detected in both cell models under hypoxic conditions. The truncated promoter constructs of the PSMD4 gene were transfected into the PC3 cell line; similarly, the basal transcriptional activities of the promoter fragments were increased in hypoxic conditions (Aydoğan Türkoğlu et al., 2015). The possible binding sites of HIF in the PSMD4 gene promoter which were obtained by bioinformatics analysis were also verified by the EMSA technique. Conclusively, hypoxic conditions led to upregulation of PSMD4 gene expression by directly binding of HIF1 transcription factors to the consensus site of HRE element in the PSMD4 promoter. The data suggest that PSMD4 could be a novel hypoxia target gene for prostate cancer therapy. 

## 2. Materials and methods

### 2.1. Material

Human prostate cell line (PC3) was obtained from Dr. Kemal Sami Korkmaz, Ege University (EBILTEM) (İzmir, Turkey) and human umbilical vein endothelial cell line (HUVEC) was obtained from Dr. Ayhan Bilir, İstanbul University (İstanbul, Turkey). All tissue culture reagents were purchased from Invitrogen (Waltham, MA, USA). The antibodies were purchased from Abcam (Cambridge, UK), Santa Cruz Biotechnology (Dallas, TX, USA), and Sigma-Aldrich (St. Louis, MO, USA). Assay reporter kits (luciferase) were sourced from Clontech (Mountain View, CA, USA). Cloning primers and EMSA primers were acquired from Macrogen (Seoul, South Korea).

### 2.2. Strategies for the cloning of human PSMD4 promoter 

PC3 genomic DNA was amplified using primers containing HindIII and XhoI for upstream region 682 bp of the translation start site PSMD4 gene (GenBank accession no. NG_029700.1). Taq DNA Polymerase (Thermo Scientific, Waltham, MA, USA) was used for the PCR reaction, and the PCR product was cloned into pGEM-T Easy vector (Promega, Madison, WI, USA) with a T: A cloning system. Truncated mutations of the human PSMD4 gene promoter were prepared using the PCR-based approach. PSMD4 promoter primers sequences are as follows: forward primer for 682 bp promoter construct, 5’-CTC GAG TAG AAA GTC ATT AGC GAT C-3’; forward primer for 326 bp promoter construct, 5’-CTC GAG GGA ATC GAC ACA GCA ACT-3’; forward primer for 150 bp promoter construct, 5’-CTC GAG TAC AAC TCC CAG AAC GCA A-3’; truncated promoter constructs reverse primer, 5’-TTC GAA CAT CTT GCC ACC TTC CTC C-3’. All constructs were sequenced (REFGEN, Ankara) using pMetLuc sequencing primer to confirm the sequence integrity. Subsequently, all constructs in pGEMT-easy were subcloned into pMetLuc reporter vector at the XhoI and HindIII restriction sites. The 682 bp, 326 bp, and 150 bp of promoter constructs were designated as pMET_PSMD4_682 (–630/+52), _PSMD4_326 (–274/+52) and _PSMD4_150 (–98/+52), respectively. 

### 2.3. Cell culture and chemically-mimicked hypoxic conditions 

PC3 and HUVEC cells were cultured in Dulbecco’s Modified Eagle Medium (DMEM) supplemented with 10% fetal calf serum (FCS) under humidified air containing 5% CO_2_ at 37 °C. Cell viability was ensured by trypan blue exclusion. For both mRNA and protein analysis, cells were seeded at 2,000,000 cells/25 cm2 flask, and cells were incubated overnight to allow cells to attach to wells. A chemical mimetic of hypoxia, CoCl_2_, was used to cause a hypoxic response in cells; 150 μM concentration was determined as the optimum dose for hypoxia. For hypoxic conditions, PC3 and HUVEC cells were treated with 150 μM final concentration of CoCl_2_ for different time intervals, namely 1, 3, 6, and 24 h (Aydogan Turkoglu and Kockar, 2016).

### 2.4. MTT assay

The viability of cells was determined by MTT assays (Aydogan Turkoglu et al., 2019). Cells were plated onto a 96-well plate briefly and incubated with 150 μM CoCl_2_ for 1–24 h. At the end of the incubation times, the cells were treated by adding MTT 20 μL (5 mg/mL) to each well and incubated at 37 °C for 4 h. Subsequently, the resultant medium was discarded and dissolved in formazan crystals of 100 μL acidified isopropanol (0.04 N HCl). The absorbance was measured with a spectrophotometer (Thermo Scientific) at 550 nm. Two independent experiments were conducted in triplicate.

### 2.5. Transient transfection of promoter constructs

Various lengths of 5’ truncated PSMD4 promoter constructs were transiently transfected into PC3 cells by the calcium–phosphate precipitation method (Aydogan Turkoglu and Kockar, 2016). SEAP plasmid was used for internal transfection efficiency control. The secreted luciferase/SEAP activities in the cell medium were determined using Ready to Glow secreted luciferase kits (Clontech). The luciferase activity was normalized to the SEAP value, and each transfection was repeated at least 3 times. Cells were treated with 150 μM CoCl_2_ after transfection for 48 h to create a chemically-mimicked hypoxic model; nontreated cells were included for each assay.

### 2.6. RNA isolation and quantitative real-time PCR (qRT-PCR)

The GeneJET™ RNA Purification Kit was used to extract total RNA according to the manufacturer’s recommendation (Thermo Scientific). Revert Aid Reverse Transcriptase (Thermo Scientific) was used to generate cDNA. mRNA expression levels of PSMD4, HIF1α, and human β2 microglobulin (hβ2) were determined with SYBR Green I Master, (Roche, Basel, Switzerland). The expression primer sequences for PSMD4 are 5’-GAA GGT GGC AAG ATG GTG TTG GAA A-3’ and 5’-TCC TTC TCA TTG TCC TCC ACT GGG CT-3’. hβ2 internal controls primers are 5’-TTT CTG GCC TGG AGG CTA TC-’3 and 5’-CAT GTC TCC ATC CCA CTT AAC T-’3. HIF-1a expression primer sequences are 5’-CCA CCT ATG ACC TGC TTG GT-’3 and 5’-TGT CCT GTG GTG ACT TGT CC-’3. A PCR reaction was achieved with 10 µL total volume; by using melting curve analysis, product specificity verification was done. The results were analyzed with the Δ-Ct method (Livak and Schmittgen, 2001). 

### 2.7. Western blot

Preparation of protein samples was performed with a RIPA buffer, and the samples were size-fractionated under reducing conditions using 10 % (w/v) polyacrylamide gels, followed by a transfer step of blotting with Immobilon-P PVDF membranes (Millipore, Burlington, MA, USA). Following the blotted membrane incubation with antibodies, bands were identified by means of X-ray–sensitive film (Kodak, Rochester, NY, USA) and an enhanced chemiluminescence detection kit (Pierce Chemical Co., Dallas, TX, USA). Image J software was used for quantitative analysis of relative protein expression of β-Actin and PSMD4 (Aydogan Turkoglu and Kockar 2016; Aydogan Turkoglu et al., 2019). 

### 2.8. Electrophoretic mobility shift assays (EMSA)

EMSA was carried out in accordance with Pierce manufacturer’s instructions (Thermo Scientific) with slight modifications (Tokay and Kockar, 2016; Aydemir et al., 2018). Oligonucleotides used as a probe were 5’GGC ACC TAC AAC TCC CAG AAC GCA ACG TGG GGG ACG GAG GCG GAA GCA GCT GGC CAA GCC GAG GT’3 and 3’CCG TGG ATG TTG AGG GTC TTG CGT TGC ACC CCC TGC CTC CGC CTT CGT CGA CCG GTT CGG CTC CA’5. Briefly, the DNA binding reaction was conducted using DNA-binding buffer and 4 μg PC3 nuclear extracts. Cells were treated with 150 µM CoCl_2_ for 24 h; these cells were used as a hypoxic nuclear extract. Samples were placed in 5% native polyacrylamide gel and subsequently transferred to nylon membranes. Detection of complexes was performed by autoradiography (Thermo Scientific). Unlabeled cold probe and HIF1α consensus oligos were employed for a competition assay. 

### 2.9. Statistical analysis

One-way analysis of variance (ANOVA) was used for statistical analysis in Minitab; P values of 0.05 or less were accepted for statistical significance of a probability (P). 

## 3. Results

### 3.1. In silico analysis and cloning of human PSMD4 promoter

**Figure 1:**
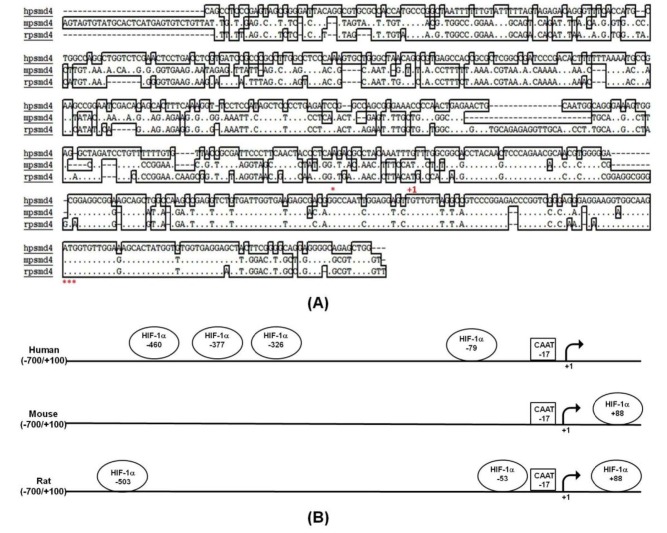
Bioinformatics analysis human, mouse and rat PSMD4 promoter. A) Multiple alignments of PSMD4 promoter (–488/+112) were performed. Asterisks in the underneath of the alignment show conserved residues. (+1) transcription start site, *ATG; translation initiation codon are indicated. B) Human, mouse, and rat PSMD4 promoters (–700/+100) are aligned, and putative HIF1a binding sites and some consensus sequences are given.

Nucleotide sequence databases for PSMD4 cDNA sequences were investigated in order to identify the putative promoter region of the PSMD4 gene (GenBank accession no. NG_029700.1). The PCR-based approach was used to clone –632/+50 bp of the PSMD4 promoter region into pGEMT-easy vector using genomic DNA. Multiple alignment analysis of 800 bp of human, mouse, and rat PSMD4 promoter sequence showed that the first 200 bp is highly conserved amongst species but there are some insertions—29bp (–488/–459) in the mouse promoter and 15 bp (–156/–141) in the rat promoter region. Pairwise alignment of human PSMD4 and mouse PSMD4 promoter regions and human PSMD4 and rat PSMD4 promoter regions show similar identity (52% identity), although mouse and rat PSMD4 regions are 80% identical (Figure 1A). Transcription factor binding search was performed to analyze the potential TF binding elements in the promoter 800 bp PSMD4; the sequence also presented the occurrence of some potential transcription factor binding sites for HIF1 (Figure 1B). MatInspector and EPT programs indicated 4 putative HREs in human PSMD4 promoter, locations –460, –377, –326, and –79. Rat and mouse PSMD4 (but not human PSMD4) possess the common HRE element located at +88 bp.

### 3.2. Verification of hypoxia and its effects on PC3 and HUVEC cell proliferation

**Figure 2:**
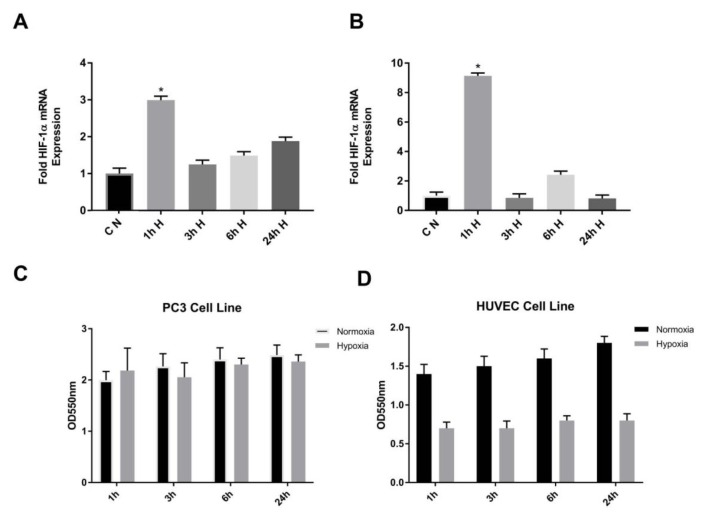
A) To determine HIF1α mRNA levels of PC3. B) HUVEC cells under normoxic (control) and hypoxic (CoCl_2_) conditions. Total RNA was isolated and then reverse transcribed and 1 μg cDNA used as a template for realtime PCR. Data are shown as relative expression of HIF1/Hb2 mRNA level. Nontreated cells were used as control. C) To investigate the effect of hypoxic conditions on cell proliferation PC3. D) HUVEC cells were treated with CoCl_2_ for hypoxia. MTT assay was performed as described above. 150 μM CoCl_2_ was used for hypoxic treatment. Nontreated cells were used as control.

Hypoxia is an environment in which oxygen is reduced. The hypoxic microenvironment is a dominant feature of prostate cancers. There are virtually no studies available on the hypoxic regulation of the PSMD4 gene in a prostate cancer model. In addition, in our previous study, the PSMD4 expression was aberrant in the PC3 and HUVEC cell lines (Aydoğan Türkoğluet al., 2018). In order to identify the hypoxic regulation of PSMD4 in PC3 cell lines, we first confirmed HIF1α overexpression in PC3 and HUVEC cells after inducing hypoxia with CoCl_2_. For this, total RNA was isolated from both the cells studied as a control and the hypoxic group. Real-time PCR analyses were performed with specific HIF1a primers. With HIF1α mRNA level in PC3 and HUVEC cells overexpression control indicated HIF1α expression was perfectly upregulated in the cells at 1 h, as shown in Figures 2A–2B.

To investigate the effect of hypoxic conditions on PC3 and HUVEC cell proliferation, the MTT test was performed. We did not obtain any statistically significant increasing or decreasing effect of cell proliferation in the PC3 cell line. A decrease in cell proliferation was observed with CoCl_2_ treatment in the HUVEC cell line (Figures 2C–2D). 

### 3.3. Effect of hypoxia on PSMD4 gene regulation

**Figure 3:**
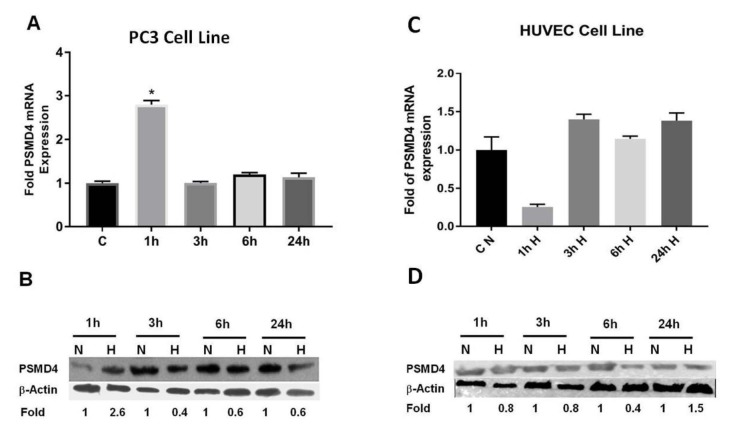
To investigate PSMD4 mRNA and protein expression in PC3 and HUVEC cell lines under hypoxic conditions, cells were treated with 150 μM CoCl2. Nontreated cells were used as control. A) PSMD4 mRNA levels were determined with a qRT-PCR, normalized to hβ2 levels in PC3 cell line. B) PSMD4 protein levels were determined with Western blot, normalized to β-actin levels in PC3 cell line. C) PSMD4 mRNA levels were taken qRT-PCR, normalized to hβ2 levels in HUVEC cell line. D) Protein levels were analyzed with Western blot, normalized to β-actin levels in HUVEC cell line.

In order to investigate hypoxic regulation on PSMD4 expression, PSMD4 mRNA expression was analyzed by qRTPCR at different time points. PSMD4 mRNA expression was increased by hypoxia at an early time point (1 h). PSMD4 expression at the protein level with hypoxia was confirmed by Western blot; we also obtained increased PSMD4 protein level in hypoxia at 1 h (Figures 3A–3B). 

**Figure 4:**
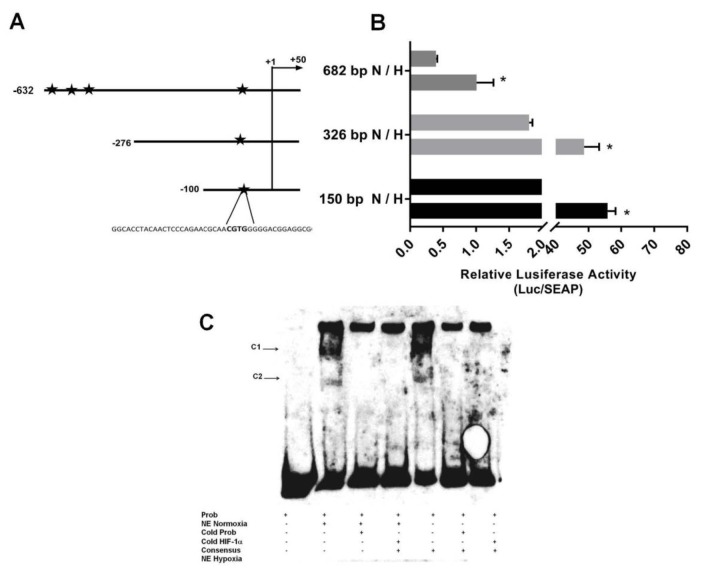
A) Schematic representation of truncated promotor constructs. Arrows point the transcription direction and numbers show the beginning and endpoints of each construct related to the transcription start site (TSS). 682 base pair promoter construct (–632/+50), 326 base pair promoter construct (–276/+50) and 150 base pair promoter construct (–100/+50) were represented by arrows. Hypoxia response elements (HREs) were indicated by a star in promoter constructs. B) For the effect of hypoxia on PSMD4 promoter activity in PC3 cells, transient transfection assay was performed using the truncated promoter constructs. 682 base pair promoter construct in normoxia and hypoxia activities were indicated by 682 bp N/H abbreviation; 326 base pair promoter construct in normoxia and hypoxia activities were indicated by 326 bp N/H abbreviation and 150 base pair promoter construct in normoxia and hypoxia activities were indicated by 150 bp N/H abbreviation. The basal activities of the promoter constructs were used for comparison. Data is the mean of three individual values ± SD. C) HIF1α protein in vitro binding to the PSMD4 promoter region was examined through EMSA. Competition to probes labeled biotin [−131/−103] were carried out with unlabeled HIF1a consensus oligonucleotide with a nuclear extract (NE) of PC3. Complexes 1 and 2 were indicated by C1 and C2.

In order to define basal promoter activity, DNA fragments corresponding to the 682 bp of 5’-flanking regions of the PSMD4 gene were cloned into the pMetLuc luciferase reporter vector. Consequently, to determine the regulatory promoter regions, 3 unique 5’deletion constructs were produced and cloned into the pMetLuc reporter plasmid. Figure 4A shows a schematic diagram of these constructs; specifically, pMET_PSMD4_682 (–630/+52), pMET_PSMD4_326 (–274/+52), and pMET_PSMD4_150 (–98/+52) Chimeric constructs, which include the 52 bp of the 5’ UTR region, were transiently transfected to PC3 cells. Transcription activities were assessed by dual-luciferase assays. SEAP activity was also determined from a transfected medium for normalization of luciferase activity of the constructs. All promoter constructs were active in PC3 cells (Figure 4B). In fact, a 150-bp fragment of pMET_PSMD4_150 (–98/+52) was adequate to trigger gene expression in this cell, as shown in Figure 4B. pMET_PSMD4_326 (–274/+52) and pMET_PSMD4_150 (–98/+52) promoter basal activities were stronger than pMET_PSMD4_682 (–630/+52) in PC3 cells. This suggests the presence of different cis-acting elements. 

### 3.4. Analysis of HRE element in PSMD4 promoter 

Hypoxia response elements (HREs) are cis-acting elements holding the consensus core sequence 5’-(A/G)CGT(G/C)(G/C)-3’, and are localized at varying positions and orientations of the coding region of numerous genes regulated by hypoxia (Marignol et al., 2005). Based on in silico analysis, the PSMD4 promoter contains many putative HIF-1 binding sites: –460, –370, –326, and –79 sites. To investigate the effect of the HIF1α protein on PSMD4 promoter activity, PC3 cells were transfected with truncated PSMD4 promoter construct pMET_PSMD4_682 (–630/+52), pMET_PSMD4_326 (–274/+52) and pMET_PSMD4_150 (–98/+52), respectively in the absence or presence of CoCl_2_.

Overinduction of HIF1α by CoCl_2_ significantly increased all of the PSMD4 promoter fragments’ activity (Figure 4B), but the highest increases were seen in the promoter construct pMET_PSMD4_326 (–274/+52) and pMET_PSMD4_150 (–98/+52). 

To identify HIF1 in vitro binding to putative PSMD4 promoter recognition sequences, an initial bioinformatics analysis of PSMD4 promoter for putative HIF1 binding sites was performed. This analysis identified 4 putative HREs in the PSMD4 promoter (Figures 1B and 4B). The luciferase experiments with the promoter fragments showed us that the hypoxic response was highest in the smallest promoter fragment (Figure 4B). Therefore, the first HRE element localized at –79 bp of PSMD4 promoter was examined in an EMSA assay. EMSA experiments were performed with biotin-labeled oligonucleotide probes in the first 150 bp of PSMD4 core promoter region (–104/–39) and normoxic/hypoxic treated PC3 nuclear extracts (Figure 4C). EMSA produced 2 DNA-protein complexes in normoxic and hypoxic conditions. However, the binding of Complex 1 in hypoxia-treated nuclear extract was stronger than the one in normoxic nuclear extract. Competitive unlabeled probes were used to evaluate the specificity of the complexes. From the competition experiment, it was found that the complexes were specific. Furthermore, biotin-labeled probes binding to PSMD4 DNA were competitively diminished by the addition of unlabeled consensus HIF1 probe (Figure 4C), which showed that HIF1 specifically binds to related regions. 

### 3.5. PSMD4 mRNA and protein expression in HUVEC cell line

We also examined whether the expression of the PSMD4 gene is induced in hypoxic HUVEC cell line. As shown in Figure 3C, the PSMD4 mRNA expression decreased as early as 1 h after hypoxic stimulation, as seen in the quantitative qRT-PCR analysis. Interestingly, the decreased expression is turned into increased PSMD4 mRNA expression by hypoxia at 3 h, 6 h, and at 24 h. The production of PSMD4 at the protein level by hypoxia was confirmed by Western blot; we also obtained decreased PSMD4 protein levels in hypoxia at 1 h, 3 h, and 6 h (Figure 3D). We only detected the increased PSMD4 protein level at 24 h. The increased expression of PSMD4 levels in the PC3 cell line was not parallel to their expression in hypoxic HUVEC. This shows cell-type–specific regulatory elements could regulate PSMD4 promoter activity in PC3 and HUVEC cell lines for promoter constructs. As a result of this regulation, mRNA and protein levels were changed in these cell lines.

## 4. Discussion

To date, researchers have focused on exploiting the hypoxic nature of tumors in order to design therapeutic strategies for overcoming hypoxic resistance (Patel and Sant, 2016). Oxygen-regulated genes are involved in numerous cellular processes including angiogenesis, glucose homeostasis, inflammation, and vascular permeability (Weidemann and Johnson, 2008). Hypoxia-inducible factor-1 (HIF-1) has been found to be the only directly oxygen-responsive DNA regulatory element regulating the expression of oxygen-responsive genes. While inactive in normoxic circumstances, reduced oxygen tension prompts HIF-1 to bind to HREs, which are found in the promoters of some oxygen-responsive genes. HREs contain the consensus core sequence 5’-(A/G)CGT(G/C)(G/C)-3’, making them cis-acting elements; they are localized at some positions and orientations of several hypoxia-regulated gene coding regions (Marignol et al., 2005).

On the other hand, a dominant feature of prostate cancers is a hypoxic environment u1d3c et al., 2019). The chemically-mimicked hypoxic environment was formed as described in our previous study (Aydogan Turkoglu and Kockar, 2016). In our study, the chemically hypoxic-mimetic agent CoCl_2_ was used to create a hypoxic environment. The time intervals were selected according to the hypoxic response given by the cells. HIF-1α mRNA level was evaluated for hypoxic response. Time intervals 1, 3, 6, and 24 h were then selected in the experimental design. The working principle of hypoxia is that proline hydroxylases inhibit their activity by binding to HIF-1α when oxygen is sufficient. The HIF-1α protein, which is unable to function, is destroyed in the proteasome. In the case of CoCl_2_, CoCl_2_ specifically inhibits proline hydroxylases that do not bind to HIF-1α and the protein becomes active. It reaches the level of oxygen deficiency and affects the transcription of hypoxia-regulated genes (Wang et al., 1993, 1995; Yuan et al., 2003; Torii et al., 2009). 

The ubiquitin–proteasome pathway has a vital part in cellular processes because it controls the expression of numerous important proteins involved in oncogenesis and cell cycle progression (Shen et al., 2013; Chen et al., 2016). In growing mammalian cells, the great majority of protein degradation is catalyzed by 26S proteasome. Numerous studies have found that PSMD4 plays a significant part in tumor progression. An overexpression of PSMD4 has been found in human colon cancer (Lin et al., 2016; Cheng et al., 2018). Furthermore, PSMD4 has been amplified and overexpressed in breast cancer, and this overexpression is correlated with low survival rates (Godek et al., 2011; Fejzo et al., 2017). PSMD4 also increases cell proliferation in HCC; there is a significant correlation between HIF1 expression and PSMD4 expression in HCC (Jiang et al., 2019). 

Regulation of PSMD4 was studied for the first time in chemically-induced hypoxic PC3 cells. In our previous studies, chemically mimicked hypoxia has been successfully applied in a different cell line (Aydogan Turkoglu and Kockar, 2016). CoCl_2_ showed no cytotoxic effect on PC3 cells, but cell death occurred in HUVEC cells. Similarly, this effect on HUVEC cells has been shown in different studies. Overexpression of HIF-1 reduced HUVEC cell viability through autophagy (Wu et al., 2015); HUVEC cell viability was also significantly affected by CoCl_2_ doses at 200–400 µM concentrations (Liang et al., 2017). 

The hypoxic condition upregulates PSMD4 gene expression at the mRNA and protein level. The regulation of transcription and translation can be differently regulated for the PSMD4 gene in PC3 cells. We think that the amount of a protein decreases when the transcription of its encoding gene is reduced, and that there are other mechanisms regulating the protein’s abundance. For example, the half-life of the protein could be decreased due to an induced rate of degradation. Another possibility is that its mRNA is more preferentially translated during the condition we analyzed at 1 h. Transcriptional activity of the PSMD4 gene show powerful basal activity in PC3 cell line. The basal activities of pMET_PSMD4_326 (–274/+52) and pMET_PSMD4_150 (–98/+52) promoter are stronger than those of pMET_PSMD4_682 (–630/+52) in PC3 cells. Cell-type–specific regulatory elements could regulate PSMD4 promoter activity in PC3 cells for promoter constructs. The longest promoter construct, pMET_PSMD4_682 (–630/+52), might have a reduced silencer region. Based on in silico analysis, the PSMD4 promoter consists of 4 putative HIF-1 binding sites: –460, –377, –326, and –79 sites. Overinduction of HIF-1a by CoCl_2_ significantly increased all of the PSMD4 promoter fragments’ activity (Figure 4B), but the highest responses are seen in the promoter construct pMET_PSMD4_326 (–274/+52) and pMET_PSMD4_150 (–98/+52). In recognizing HIF1-dependent regulation of the PSMD4 core promoter, EMSA trials with probes containing biotin-labeled oligonucleotide reside in −131/−103 promoter were performed in nuclear extracts from PC3 cells. The results indicated that HIF1a can bind to the corresponding region in prostate cancer cells. 

In conclusion, this study shows that hypoxia regulates PSMD4 gene expression along with transcriptional activity regulated by the HIF1a transcription factor. This finding is important for the development of prostate cancer therapy that uses hypoxic conditions. Our findings show hypoxia-regulated PSMD4 expression in prostate cancer and give valuable information for designing novel treatments. The study delivers new data on PSMD4 regulation by HIF1a in the prostate cancer cell line.
